# Fault Diagnosis of Wind Turbine Gearbox Based on Modified Hierarchical Fluctuation Dispersion Entropy of Tan-Sigmoid Mapping

**DOI:** 10.3390/e26060507

**Published:** 2024-06-11

**Authors:** Xiang Wang, Yang Du

**Affiliations:** 1 School of Energy and Power Engineering, Nanjing Institute of Technology, Nanjing 211167, China; wangxiang@njit.edu.cn; 2 School of Electrical Engineering, Nanjing Institute of Technology, Nanjing 211167, China

**Keywords:** gear box, fault diagnosis, tan-sigmoid mapping, modified hierarchical fluctuation dispersion entropy, support vector machine

## Abstract

Vibration monitoring and analysis are important methods in wind turbine gearbox fault diagnosis, and determining how to extract fault characteristics from the vibration signal is of primary importance. This paper presents a fault diagnosis approach based on modified hierarchical fluctuation dispersion entropy of tan-sigmoid mapping (MHFDE_TANSIG) and northern goshawk optimization–support vector machine (NGO–SVM) for wind turbine gearboxes. The tan-sigmoid (TANSIG) mapping function replaces the normal cumulative distribution function (NCDF) of the hierarchical fluctuation dispersion entropy (HFDE) method. Additionally, the hierarchical decomposition of the HFDE method is improved, resulting in the proposed MHFDE_TANSIG method. The vibration signals of wind turbine gearboxes are analyzed using the MHFDE_TANSIG method to extract fault features. The constructed fault feature set is used to intelligently recognize and classify the fault type of the gearboxes with the NGO–SVM classifier. The fault diagnosis methods based on MHFDE_TANSIG and NGO–SVM are applied to the experimental data analysis of gearboxes with different operating conditions. The results show that the fault diagnosis model proposed in this paper has the best performance with an average accuracy rate of 97.25%.

## 1. Introduction

Wind turbines have become one of the major developments in the global renewable energy sector [1].They are widely applied across various countries and regions as a significant component of the power supply [2,3]. Wind turbines are subjected to unstable working conditions, as well as exposed to high wind speeds, extreme temperatures, humidity, and corrosive climates for long periods of time [4,5,6]. These factors can cause mechanical fatigue and component aging, which can lead to wind turbine failures. The gearbox, as a complex wind turbine component, is subject to high torque and changing wind loads, which is a leading cause of wind turbine malfunction [7]. This paper introduces a data-driven intelligent diagnostic approach for identifying vibration faults in wind turbine gearbox vibration signals.

Complex nonlinear vibration signals in gearboxes can be caused by various failure factors, such as damage gears, meshing problems and poor lubrication of gears [8,9]. Linear signal analysis methods will miss important feature information in fault diagnosis, while the use of entropy value analysis methods can better deal with nonlinear signals [10,11]. The common entropy methods such as sampling entropy [12], permutation entropy [13], fuzzy entropy [14] and dispersion entropy (DE) [15] are often used to extract features from signals. Several scholars have proposed multiscale entropy in order to better capture and describe the complex structure and information in a signal. Multiscale entropy provides a more comprehensive information analysis, which considers the information changes under different time scales. Jin et al. [16] proposed a technique for diagnosing bearing faults utilizing a composite multivariate multiscale fuzzy entropy that has been refined through segmentation and a convolutional neural network. Song et al. [17] introduced a defect diagnosis technique that employs variational modal decomposition, multiscale entropy, and the Adaboost algorithm. Zhang et al. [18] performed adaptive decomposition of vibration signals using fast ensemble empirical modal decomposition to calculate different scales of DE applied to bearing defect diagnosis. Nevertheless, the above methods still have several associated problems.

(1) The entropy methods mentioned above have inherent flaws. Sampling entropy can be a complex and time-consuming process, making it unsuitable for real-time monitoring [19]. Fuzzy entropy is also inefficient to calculate [20], while permutation entropy fails to take into account the relationship of magnitudes among amplitudes [21]. Although DE overcomes these drawbacks, it only considers the absolute nature of the magnitude and cannot assess the volatility of the signal [22].
(2) Multiscale entropy disregards the high-frequency details found within time series; it mainly assesses the low-frequency information [23].

This paper introduces fluctuation dispersion entropy (FDE) to solve problem 1 and the hierarchical decomposition of time series as a solution to problem 2, in order to eliminate the interference of the above problems.

Azami et al. [24] introduced the concept of FDE. This entropy measure considers the volatility of the series, which is more robust to the presence of underlying trends in the time series. The method is both computationally efficient and stable as it reduces all possible dispersion patterns for the same parameters.

Jang et al. [25] proposed hierarchical entropy as a means of viewing signals from a multiscale perspective through hierarchical decomposition. They achieved this by constructing a hierarchical method of high- and low-frequency operators. Hierarchical processing takes into account all frequency components in the signal, leading to a more comprehensive and accurate assessment compared to coarse-grained multiscale processing.

However, hierarchical processing still has some shortcomings. The sequence length is reduced by half with each additional decomposition layer. Shorter time series do not provide sufficient information to accurately reflect the characteristics of the primary signal, leading to a decrease in the stability of the calculation results and a potentially large margin of error. As a consequence, the accuracy and reliability of the time series are affected by the traditional hierarchical treatment. Li et al. [26] proposed an improved stratification method to address this issue. The drawbacks of the traditional hierarchical approach are significantly overcome by defining different averaging operators at different levels through moving average and moving difference in the hierarchical process. The improved method ensures that the approach is no longer affected by the length of data, and its calculation accuracy is greatly improved.

Mapping the time series into different classes is a crucial part of the FDE, and traditional entropy algorithms typically utilize the NCDF to achieve this function [27]. However, the wind turbine working environment has unique characteristics that often contaminate the gearbox vibration signal with significant noise [28]. This noise can adversely affect subsequent wind turbine fault diagnosis. Common transfer functions include the log-sigmoid (LOGSIG), TANSIG, and purelin functions [29]. Among these, the TANSIG function is less sensitive to small fluctuations in the input, resulting in greater noise resistance [30]. Therefore, this paper proposes replacing NCDF in MHFDE with TANSIG mapping.

Intelligent learning algorithms, especially deep learning methods, have the ability to comprehensively investigate the relationships between features, with powerful expressive and classification capabilities. Therefore, they are widely used in various fields. In the field of fault diagnosis, classification tasks in the diagnostic process commonly use learning algorithms such as support vector machine (SVM) [31], decision trees [32], random forests [33], and neural networks [34]. Compared to other intelligent learning algorithms, SVM finds the optimal hyperplane by maximizing the spacing between categories, which gives it a better ability to generalize to unseen data, making it highly accurate when dealing with unknown data. Additionally, it is also relatively computationally efficient for small sample datasets. SVM parameters are typically optimized due to the effects of overfitting and underfitting, which can enhance classification accuracy. Dehghani et al. [35] proposed the northern goshawk optimization (NGO) algorithm in 2021. The NGO algorithm emulates the northern goshawk’s hunting procedure and is characterized by rapid convergence and strong optimization capabilities [36]. This paper utilizes the NGO to optimize the kernel function parameters g and penalty coefficient c of SVM.

This paper proposes a methodology for defect diagnosis in wind turbine gearboxes based on MHFDE_TANSIG and NGO–SVM. Firstly, an improved hierarchical method is used to reconstruct the subsequence. Then, the traditional DE is replaced by FDE and the NCDF is replaced by the TANSIG function. It is used to construct the feature matrices of different state signals of the gearbox. Finally, NGO–SVM is employed for classification and identification in order to achieve intelligent diagnosis of various gearbox faults. The experimental results demonstrate that the approach presented in this article can proficiently detect the faults with a certain level of superiority.

## 2. Basic Principle

### 2.1. Modified Hierarchical Fluctuation Dispersion Entropy of Tan-Sigmoid Mapping Method

#### 2.1.1. Fluctuation Dispersion Entropy of Tan-Sigmoid Mapping

The following are the steps involved in calculating FDE_TANSIG:

Step 1. The TANSIG function maps the original signal $x=\{x_{j},j=1,2,\ldots ,N\}$ , which is of length N, to $y=\{y_{j},j=1,2,\ldots ,N\},y_{j}\subset (0,1)$ .

(1) $$y=\frac{2}{1+e^{-2x}}-1$$

Step 2. A linear transformation is employed to map the variable *y* into the specified range $\left[1,2,\ldots ,c\right]$ :

(2) $$z_{j}^{c}=round(cy_{i}+0.5)$$

where “round” refers to the rounding function, and *c* represents the number of categories.

Step 3. The specific calculation process for the embedding vector is as follows:

(3) $$\begin{array}{l} z_{i}^{m,c}=\left\{z_{i}^{m},z_{i+d}^{m},\ldots ,z_{i+(m-1)d}^{m}\right\}\\ i=1,2,\ldots ,N-(m-1)d \end{array}$$

where *m* is the number of embedding dimensions; and *d* is the time delay;

Step 4. Determination of the dispersion pattern $\pi_{v_{0}v_{1}\ldots v_{m-1}}(v=1,2,\ldots ,c)$ . If $z_{i}^{c}=v_{0}$ , $z_{i+d}^{c}=v_{1},\ldots ,$ $z_{i+(m-1)d}^{c}=v_{m-1}$ , and $z_{i}^{m,c}$ z represents the dispersion pattern $\pi_{v_{0}v_{1}\ldots v_{m-1}}$ ;

Step 5. Determine the probability that each dispersion pattern $\pi_{v_{0}v_{1}\ldots v_{m-1}}$ exists:

(4) $$P(\pi_{v_{0}v_{1}\ldots v_{m-1}})=\frac{Num(\pi_{v_{0}v_{1}\ldots v_{m-1}})}{N-(m-1)d}$$

where $Num(\pi_{v_{0}v_{1}\ldots v_{m-1}})$ is $z_{i}^{m,c}$ mapping to $\pi_{v_{0}v_{1}\ldots v_{m-1}}$ number of individuals.

Step 6. The definition of information entropy specifies that the FDE_TANSIG of a signal *x* is given by

(5) $$`FDE\_TANSIG(x,m,c,d)=-\sum_{\pi =1}^{{\left(2c-1\right)}^{m-1}}P(\pi_{v_{0}v_{1}\cdots v_{m-1}})\cdot \ln\left(P(\pi_{v_{0}v_{1}\cdots v_{m-1}})\right)$$

#### 2.1.2. Modified Hierarchical Fluctuation Dispersion Entropy of Tan-Sigmoid Mapping

To calculate MHFDE_TANSIG for a specific time series *x*, follow these steps:

Step 1. Define two properties of the operator $Q_{0}$ and $Q_{1}$ as

(6) $$Q_{0}(x)=\frac{x(2j)+x(2j+1)}{2},Q_{1}(x)=\frac{x(2j)-x(2j+1)}{2},j=0,1,\cdots ,2^{n-1}$$

where $2^{n-1}$ is the length of the operator, n is a positive integer, and $Q_{0}(x)$ and $Q_{1}(x)$ represent the low-frequency and high-frequency components extracted for the previous layer of the signal, respectively.

Step 2. The matrix form of the kth layer operator $Q_{j}^{k}$ should be defined as follows when j equals 0 or 1:

(7) $$Q_{j}^{k\;\prime }={\left[\begin{array}{cccccccc} \frac{1}{2} & \underset{2^{k-1}-1}{\underset{︸}{0\cdots 0}} & \frac{{\left(-1\right)}^{j}}{2} & 0 & \cdots & 0 & 0 & 0\\ 0 & \frac{1}{2} & \underset{2^{k-1}-1}{\underset{︸}{0\cdots 0}} & \frac{{\left(-1\right)}^{j}}{2} & \cdots & 0 & 0 & 0\\ \cdots & & & & & & & \\ 0 & 0 & 0 & 0 & \cdots & \frac{1}{2} & \underset{2^{k-1}-1}{\underset{︸}{0\cdots 0}} & \frac{{\left(-1\right)}^{j}}{2} \end{array}\right]}_{\left(l-2^{k}+1)\times (l-2^{k-1}+1\right)}$$

Step 3. It is necessary to iteratively use the $Q_{j}^{k}$ operator defined above to calculate the hierarchical component $x_{k,e}$ for each layer during the hierarchical decomposition. Additionally, a vector $\left[r_{k},r_{k-1},\cdots ,r_{1}\right]$ and an integer value $q=\sum_{p=1}^{k}2^{k-p}r_{p}$ must be defined, where $\left\{r_{p},p=1,2,\cdots ,k\right\}\in \left\{0,1\right\}$ denotes the averaging or differencing operator for the p-th layer.

Thus, the stratification component of the q-node on the k-th layer can be represented as

(8) $$x_{k,q}=Q_{r_{k}}^{k}\times Q_{r_{k-1}}^{k-1}\times \cdots \times Q_{r_{1}}^{1}\times x$$

Step 4. Calculate FDE_TANSIG of the subsequence $x_{k,q}$ following the steps in Section 2.1.1. The final formula is

(9) $$MHFDE\_TANSIG\left(x,k,m,c,d\right)=FDE\_TANSIG\left(x_{k,q},m,c,d\right)$$

**Figure 1:**
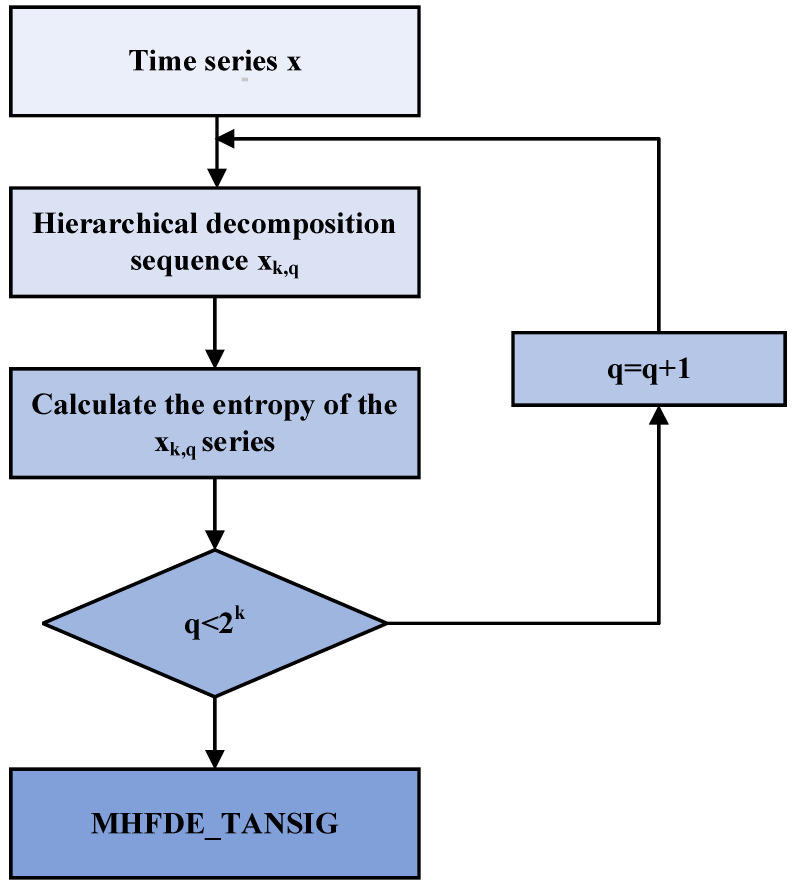
Flowchart of MHFDE_TANSIG.

Figure 1 displays the MHFDE_TANSIG flowchart.

#### 2.1.3. Parameters Select

From the definition of MHFDE_TANSIG in Section 2.1.2, it can be seen that the computational results of this method are affected by a number of factors, including the number of decomposition layers *k*, time delay *d*, embedding dimension *m*, classification class c, and the time series *x* itself. Furthermore, the length of time series *l* exerts a direct influence on the time series *x*. The selection of optimal parameter values can result in enhanced performance in entropy value calculations [26].

In this study, the configuration of the MHFDE_TANSIG requires manual adjustment of five essential parameters: decomposition layers *k*, the length of time series *l*, time delay d, embedding dimension *m*, and classification class *c*. 

**Figure 2:**
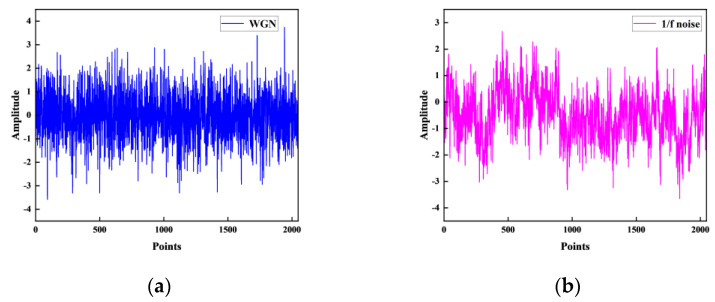
Test signals: (**a**) WGN; (**b**) 1/f noise.

The test signals used in this study are white Gaussian noise (WGN) and 1/f noise. WGN is a random signal with a Gaussian distribution that is smooth in frequency, while 1/f noise is not smooth in frequency and its power spectral density has a 1/f relationship with frequency. Figure 2 illustrates examples of WGN and 1/f noise.

The experiments described in this paper were conducted on a computer running MATLAB R2022b, which was equipped with a 12th Gen Intel(R) Core(TM) i5-12500H 2.5 GHz processor (Intel, Santa Clara, CA, USA), 16.0 GB RAM, and Windows 11 operating system. Fifty WGN and 1/f noise samples were configured as test signals to calculate the MHFDE_TANSIG values under different parameters and their runtime lengths were counted, thus evaluating the performance under different parameters.

(1) Decomposition layers *k*

The decomposition layer, denoted by *k*, represents the number of layers of decomposition in the modified hierarchical processing. In general, this value is typically between 1 and 5 [37].

**Figure 3:**
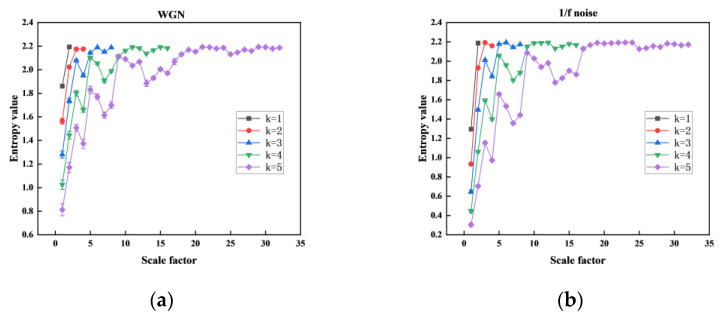
Different *k* on MHFDE_TANSIG: (**a**) WGN; (**b**) 1/f noise.

We recorded the MHFDE_TANSIG at various numbers of decomposition layers *k* in Figure 3 and counted the computation time of the entropy value of each layer at different numbers of decomposition layers *k* as shown in Table 1. This is used to assess the impact of *k* on the stability and properties of MHFDE_TANSIG. The remaining parameters of MHFDE_TANSIG are as follows: *l* = 2048; *m* = 2; *c* = 5; *d* = 1.

According to Figure 3, it can be concluded that the stability of entropy value decreases as the number of decomposition layers *k* increases, resulting in a significant reduction in computational efficiency. Conversely, the decomposed signal sequence will lack sufficient detail to obtain hierarchical components from low to high frequency if *k* is too low. As shown in Table 1, excessive number of *k* leads to computational inefficiency.

Therefore, a value of *k* = 3 is recommended for comprehensive consideration.

From the definition of hierarchical processing in Section 2.1.2, the value of the scale factor is determined by the number of decomposition levels *k*. When *k* = 3, the number of scale factors is $2^{3}=8$ .

The eight scales resulting from the three-layer decomposition of the original signal represent the components of the original signal in different frequency ranges. The subsequence under multiple scale factors after layering can more fully reflect the characteristics of the original signal under multiple scales compared to the original signal, and can be evaluated more comprehensively and accurately.

(2) The data length *l*

Subsequently, the effect of signal length *l* on the performance of MHFDE_TANSIG calculation is discussed. Signal lengths that are either excessively large or excessively small can have a detrimental impact on the efficacy of entropy calculations [38]. Therefore, the values of *l* in this study are 512, 1024, 2048, 4096, and 8192.

**Figure 4:**
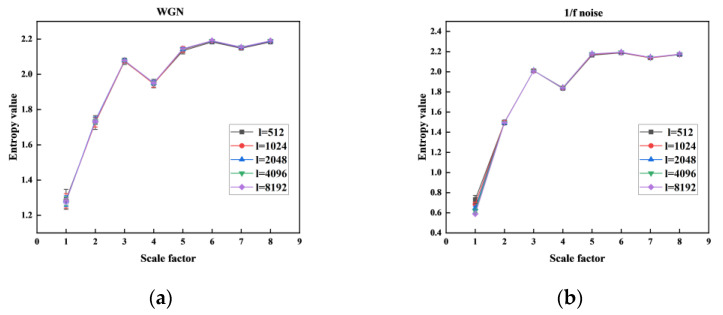
Different *l* on MHFDE_TANSIG: (**a**) WGN; (**b**) 1/f noise.

The MHFDE_TANSIG performance is tested by analyzing two noise signals for different lengths *l* of the time series, as shown in Figure 4, to investigate the effect of length. We also counted the computation time of the entropy value of each layer at different data lengths *l,* as shown in Table 2. The remaining parameters of MHFDE_TANSIG are as follows: *k* = 3; *m* = 2; *c* = 5; *d* = 1.

Figure 4 shows that the MHFDE has a high degree of overlap on most scales, indicating insensitivity to data length. However, there is some discrepancy in both the mean and error of entropy when the scale factor is 1. Table 3 shows the entropy coefficient of variation (CV) for different data lengths at a scale factor of 1.

Table 3 shows that CV decreases as data length increases, indicating that MHFDE may be less stable on certain scales with lower data length. As shown in Table 2, excessive data length *l* leads to computational inefficiency.

Therefore, a value of *l* = 2048 is recommended for comprehensive consideration.

(3) Time delay *d*

The time delay *d* in the definition of FDE denotes the time required to reconstruct the phase space, which takes values in the range of 1 to 5.

**Figure 5:**
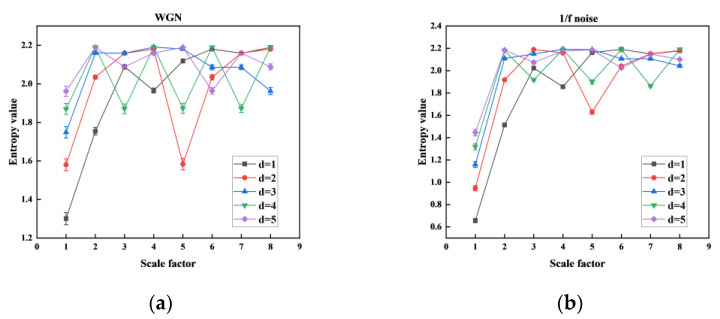
Different *d* on MHFDE_TANSIG: (**a**) WGN; (**b**) 1/f noise.

The correlation performance of MHFDE_TANSIG was validated using two noise signals with different time delays *d* as shown in Figure 5, and the computation time was statistically calculated as shown in Table 4. The remaining parameters of MHFDE_TANSIG are as follows: *l* = 2048; *m* = 2; *c* = 5; *k* = 3.

As demonstrated in Figure 5 and Table 4, there are no significant advantages or disadvantages of MHFDE with varying time delays *d*, and there is no significant difference in computation time. According to [39], certain signal frequency information may be disregarded when *d* is greater than 1 and the entropy value becomes less stable as it increases. 

Therefore, a value of *d* = 1 is recommended for comprehensive consideration.

(4) Embedding dimension *m*

The embedding dimension *m* is the dimension of the reconstructed phase space in the definition of FDE. It typically takes values in the range of 2 to 6 [37].

**Figure 6:**
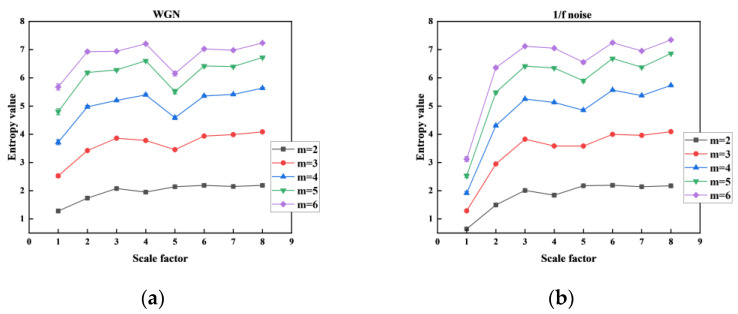
Different *m* on MHFDE_TANSIG: (**a**) WGN; (**b**) 1/f noise.

The correlation properties of MHFDE_TANSIG were validated using two noise signals with different m in Figure 6, and the computation time was statistically calculated as shown in Table 5. The remaining parameters of MHFDE_TANSIG are as follows: *l* = 2048; *k* = 3; *c* = 5; *d* = 1.

Figure 6 shows that the entropy value increases while the stability decreases as *m* increases. Stability of the entropy curve is greatest when *m* is at its minimum. Table 5 demonstrates a significant difference in computation time under different embedding dimensions, with the fastest computation efficiency when *m* is the smallest.

Therefore, a value of *m* = 2 is recommended for comprehensive consideration.

(5) Classification class *c*

The value of the classification category *c* indicates the number of dispersion patterns present in the definition of FDE. This value is typically within the range of 3 to 7 [37].

**Figure 7:**
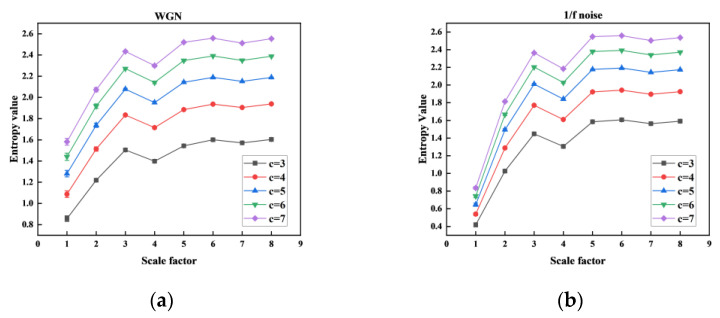
Different *c* on MHFDE_TANSIG: (**a**) WGN; (**b**) 1/f noise.

The correlation performance of MHFDE_TANSIG was validated using two noise signals with different classification class *c* as shown in Figure 7, and the computation time was statistically calculated as shown in Table 6. The remaining parameters of MHFDE_TANSIG are as follows: *l* = 2048; *k* = 3; *m* = 2; *d* = 1.

From Figure 7 and Table 6, there is no specific category with a significantly superior entropy value and computation time. However, the definition of classification class in MHFDE_TANSIG states that the value of *c* indicates the number of dispersion patterns present, assuming the other parameters are fixed. A smaller *c* value could make it challenging to differentiate between various signal classes, whereas a value that is too large could result in reduced noise immunity.

The value of *c* = 5 is recommended for comprehensive consideration to ensure a reliable trade-off between statistical measures and noise immunity performance.

#### 2.1.4. Comparison of Different Entropy Methods Based on WGN and 1/f Noise

**Figure 8:**
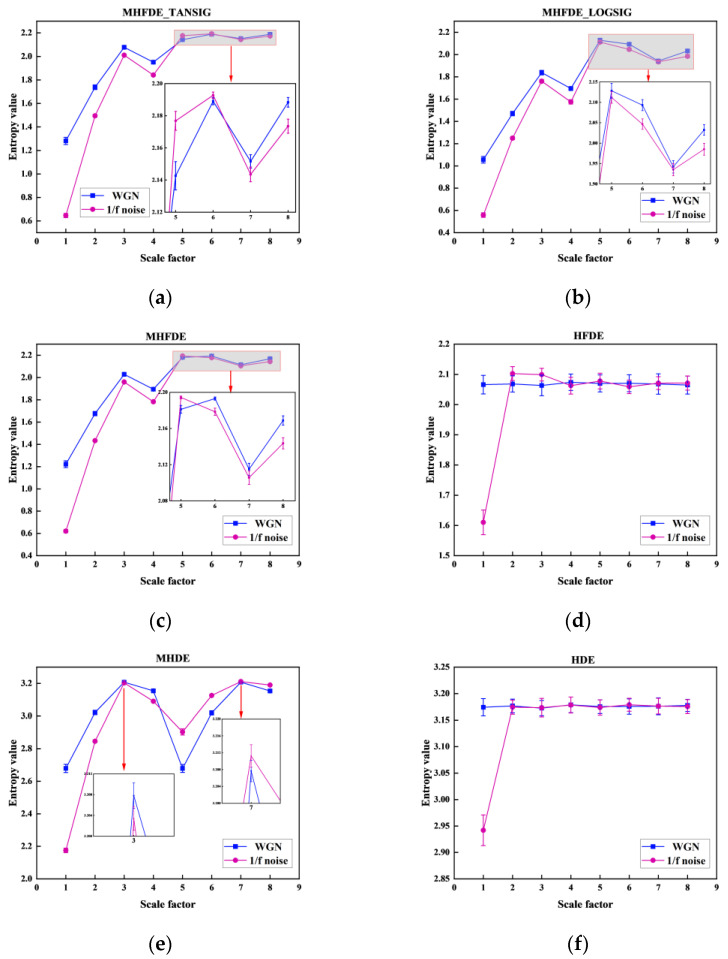
Different types of entropy value under the test signals. (**a**) MHFDE_TANSIG; (**b**) MHFDE_LOGSIG; (**c**) MHFDE; (**d**) HFDE; (**e**) MHDE; (**f**) HDE.

The results of MHFDE_TANSIG were compared with those of the unimproved entropy methods using the test signals in Figure 8. The parameters and mapping functions of various entropy algorithms are detailed in Table 7.

FDE shows a higher differentiation of different signals compared to DE from the comparison of (c) and (e) (or (d) and (f)) in Figure 8. The comparison of (c) and (d) (or (e) and (f)) in Figure 8 illustrates that the modified hierarchical processing has lower error values and higher entropy stability compared to the traditional hierarchization. None of the three types of methods show crossover in the first four scales based on different mapping functions by comparing (a), (b), and (c) in Figure 8. MHFDE_TANSIG has almost no overlapping parts in scales 5–8. However, MHFDE has a significant overlap in scale 7, and MHFDE_LOGSIG has a significant overlap in both scales 5 and 7.

### 2.2. Northern Goshawk Algorithm Optimized Support Vector Machine

#### 2.2.1. Support Vector Machine

The SVM learning algorithm uses statistical studies and minimization of structural risk to determine an optimal hyperplane that both correctly classifies the samples and maximizes their spacing [40].

The choice of kernel function is paramount for the category capabilities of SVM. The radial basis function requires fewer parameters and exhibits superior performance in classification tasks in contrast to alternative kernel functions [41]. Below is a definition of the function:

(10) $$f\left(x_{i},x_{j}\right)=\exp\left\{-\frac{∥x_{i}-x_{j}{∥}^{2}}{2g^{2}}\right\}$$

where g is a parameter that measures the complexity of the Gaussian kernel function.

In the radial basis function, c and g determine the ability to generalize the model. c indicates the preference weights for the two metrics (interval size, categorization accuracy) in the direction of adaptation and optimization. g represents the arrangement of the sequence once it has been mapped to a new feature space. The support vector machine encounters issues with extended training periods and poor accuracy when tackling issues related to multiple classifications. The findings of the classification are frequently convoluted when handling data with repeating characteristic. Currently, mature kernel function parameters are picked using subjective human experience, and, thus, feature some randomness. It is imperative to ensure that kernel functions possess suitable forms and parameters when dealing with problems in various domains.

The population optimization algorithm exhibits high efficacy and excellent optimization effects when applied in optimizing support vector machine parameters. As a result, it is extensively used in supporting vector machine algorithm optimization. The northern goshawk optimization algorithm demonstrates rapid convergence and formidable optimization abilities. This technique can assist the kernel function in finding the optimal parameter when combined with the SVM algorithm. It performs a critical role in enhancing the training speed and diagnostic accuracy of the SVM algorithm for various fault vibration signals of wind turbine gearboxes.

#### 2.2.2. Northern Goshawk Optimization Algorithm

The mathematical model used by the NGO algorithm to model the identification and pursuit of prey by northern goshawks can be succinctly defined as follows [35]:

Phase 1. Global search

During phase 1, the goshawk locks onto prey at random before launching a rapid attack. This stage can be considered a global search and can be described using the following mathematical model:

(11) $$x_{i,j}^{new,P1}=\left\{\begin{array}{cc} x_{i,j}+r\left(p_{i,j}-Ix_{i,j}\right), & F_{P_{i}}<F_{i},\\ x_{i,j}+r\left(x_{i,j}-p_{i,j}\right), & F_{P_{i}}\geq F_{i}, \end{array}\right.$$

(12) $$X_{i}=\left\{\begin{array}{cc} x_{i}^{new,P1}, & F_{i}^{new,P1}<F_{i},\\ \;\;\;x_{i}, & F_{i}^{new,P1}\geq F_{i}, \end{array}\right.$$

Phase 2. Local search

During phase 2, the prey will make an effort to flee if the goshawk is in close proximity to its prey. The goshawk’s pursuit of the prey will persist to prevent its escape; therefore, a local search strategy is employed. The mathematical model for this phase is as follows:

(13) $$x_{i,j}^{new,P2}=x_{i,j}+R\left(2r-1\right)x_{i,j}$$

(14) $$R=0.02\left(1-\frac{t}{T}\right)$$

(15) $$X_{i}=\left\{\begin{array}{cc} X_{i}^{new,P2}, & F_{i}^{new,P2}<F_{i}\\ \;\;\;X_{i}, & F_{i}^{new,P2}\geq F_{i} \end{array}\right.$$

## 3. Experimental Analysis

### 3.1. Data Acquisition

**Figure 9:**
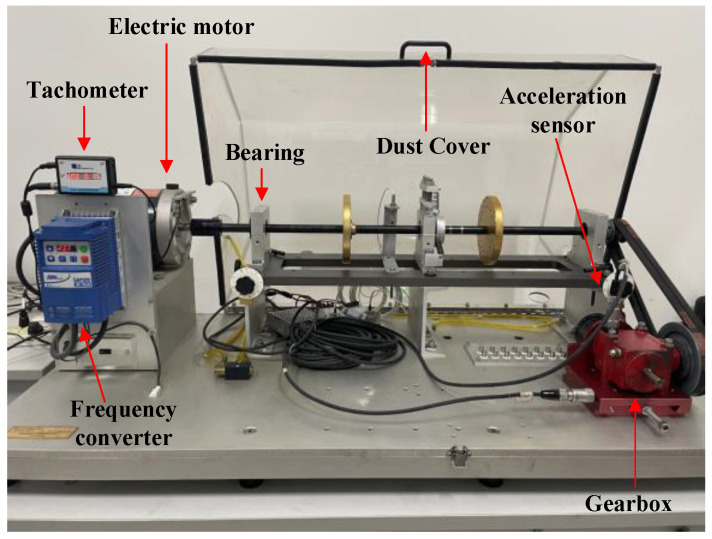
Mechanical failure simulation experiment system.

**Figure 10:**
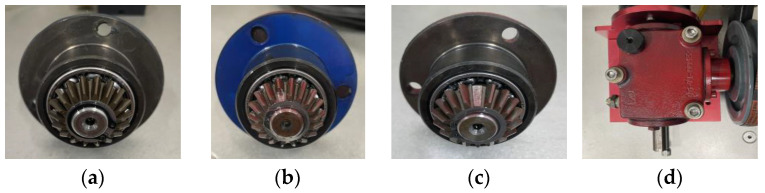
Four states of gear: (**a**) normal state; (**b**) broken tooth fault; (**c**) missing tooth fault; (**d**) surface wear fault.

The Mechanical Failure Simulation Experiment System (MFS), produced by SQI, offers detailed data on category four gearbox failures. This system is adept at replicating a variety of typical malfunctions found in mechanical equipment. It features a modular test stand, specifically engineered for simulating prevalent gear and bearing failures observed in wind turbine drive systems, thus ensuring both power and reliability. Refer to Figure 9 for an illustration of the system’s primary elements, which include an elaborate test rig for simulating mechanical failures and devices for data gathering. Figure 10 shows the gear fault diagnosis research kit, which includes normal state (NOR), missing tooth fault (MTF), broken tooth fault (BTF), and surface wear fault (SWF).

**Figure 11:**
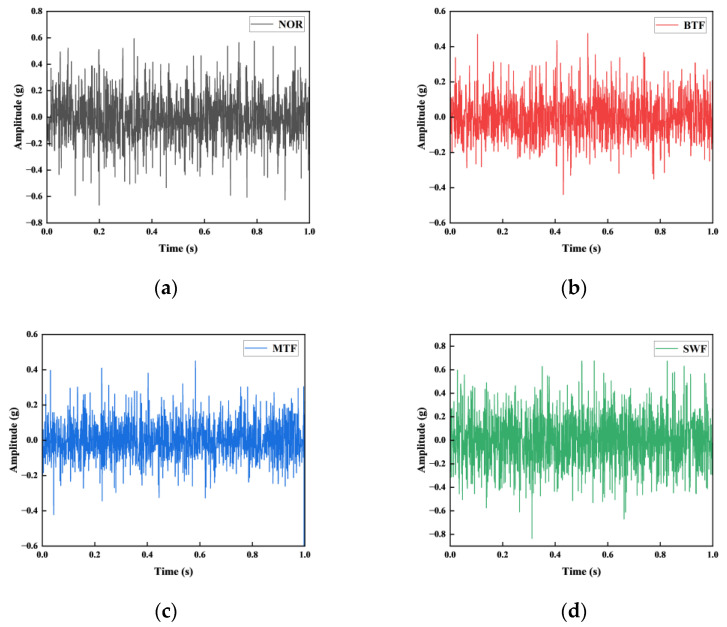
Waveform in four states: (**a**) normal state; (**b**) broken tooth; (**c**) missing tooth; (**d**) surface wear.

The motor speed was set to 1750 rpm, and the vibration indications obtained from the sensors along the y-axis on the planetary gear were chosen to run under no load during the experiment. There were four operating conditions tested: NOR; MTF; BTF; and SWF. There are 200 samples in the dataset divided into 4 groups, and each group contains 50 subsamples of 2048 sampling points each. The set of samples for each fault state is segregated into two categories: 35 samples designated for training and 15 for testing in fault diagnosis scenarios (see Table 8). Figure 11 illustrates the waveforms of the vibration indications of the gearbox under four distinct operating conditions. The horizontal coordinate indicates the duration of the captured clip in seconds s, and the vertical coordinate shows the vibration acceleration of the gearbox in the unit of gravity acceleration g in Figure 11.

### 3.2. Feature Extraction 

**Figure 12:**
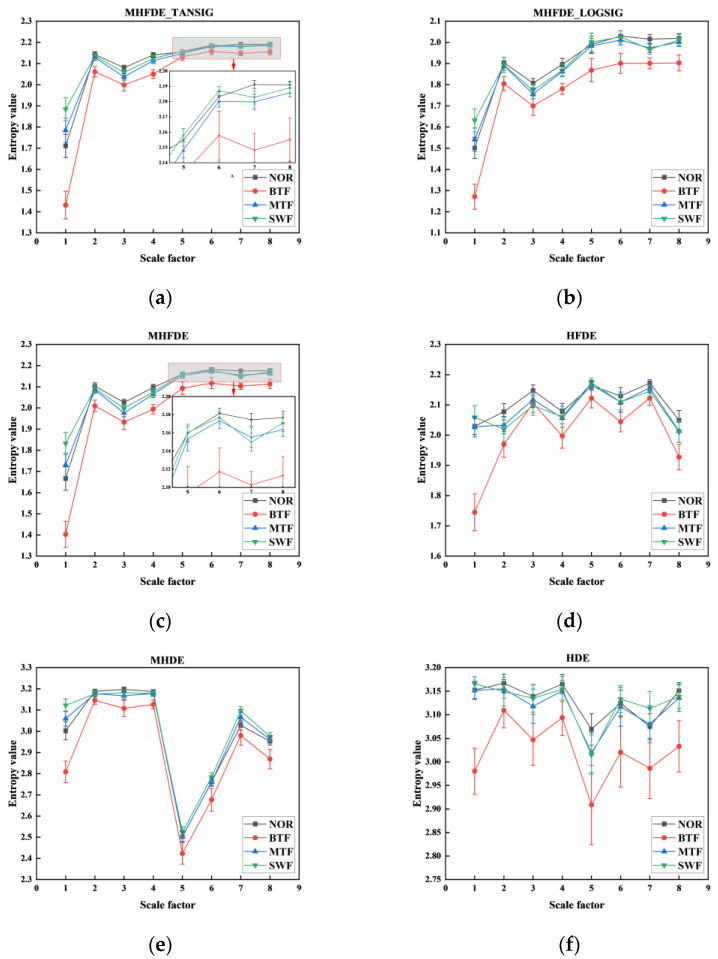
Different types of entropy value. (**a**) MHFDE_TANSIG; (**b**) MHFDE_LOGSIG; (**c**) MHFDE; (**d**) HFDE; (**e**) MHDE; (**f**) HDE.

The MHFDE_TANSIG and other entropy values for the experimental dataset of 200 samples were calculated as shown in Figure 12. Figure 6 in Section 2.1.4 displays the parameters and mapping function of different entropy algorithms.

It is evident that the various mappings of MHFDE are inconsistent in distinguishing gearbox conditions upon comparing MHFDE_TANSIG, MHFDE_LOGSIG, and MHFDE. There is partial overlap on different scales alternately, and the error is low when using the TANSIG or NCDF mapping functions in MHFDE. However, the broken lines undergo more obvious aliasing when the mapping function is LOGSIG, indicating that this mapping is less effective than TANSIG and NCDF in dealing with the gearbox signal. MHDE, HDE, and HFDE exhibit varying degrees of overlap and fluctuation in broken lines across different scales. The HDE algorithm, in particular, has a significantly high error rate. This highlights the need for optimizing feature entropy expression performance based on fluctuating dispersion entropy and improved hierarchical processing.

### 3.3. Intelligent Diagnosis

As a first step, we use the northern goshawk algorithm to optimize the two key parameters c and g of SVM, where c is optimized in the range [0.01, 10], g is optimized in the range [1, 800], and screening iterations are set to 20.

We compare particle swarm optimization (PSO), genetic algorithm (GA), and NGO to demonstrate the superiority of the NGO algorithm in optimizing SVM classification. The population size and iteration number of each algorithm are 5 and 20, respectively, and the fitness function is minimizing local sample entropy.

**Figure 13:**
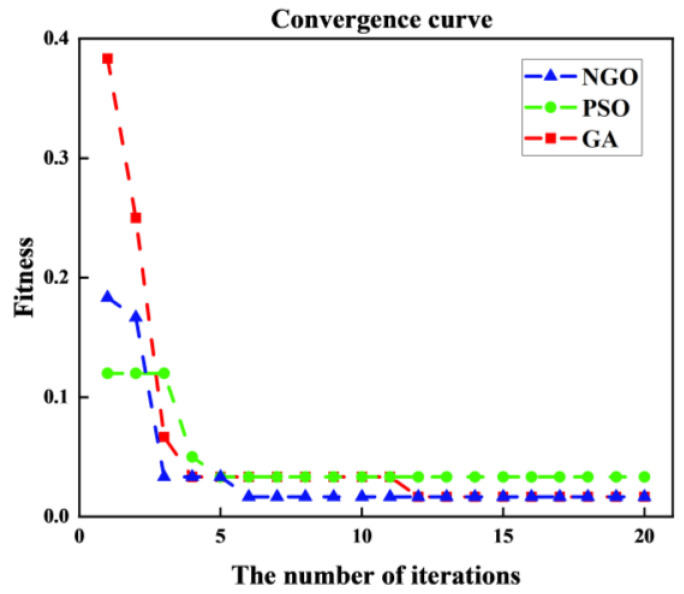
Three algorithms optimize the iteration curve.

Figure 13 displays the optimization iteration curves for the three algorithms.

**Figure 14:**
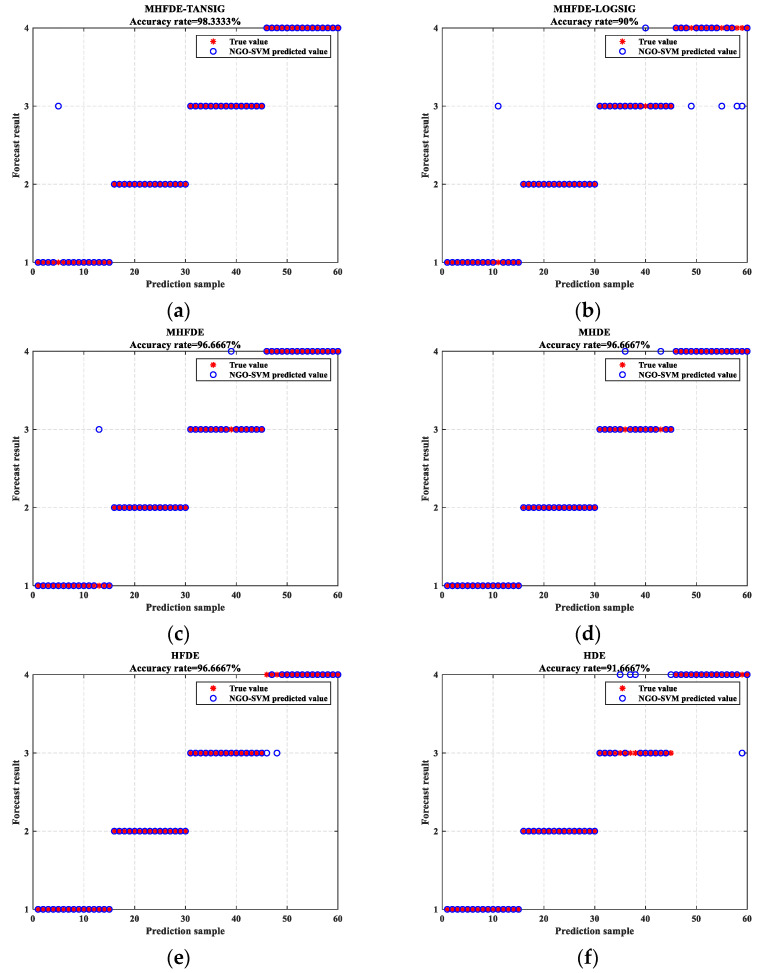
SVM test set diagnostic results. (**a**) MHFDE_TANSIG; (**b**) MHFDE_LOGSIG; (**c**) MHFDE; (**d**) HFDE; (**e**) MHDE; (**f**) HDE.

Figure 13 shows that the proposed optimum is reached after six iterations and its optimal solution is superior to the other two algorithms, proving the efficiency and performance of NGO in the optimization process. NGO-optimized SVM parameters for c and g are 2.037 and 1.485, respectively. NGO–SVM is used to classify defect features derived from different entropy methods. The diagnostic results of the SVM test set for intelligent diagnostic models are shown in Figure 14. The vertical coordinate in Figure 14 represents the forecast result, which is the classification of the prediction set data after the optimized model has been trained on the training set. Furthermore, the values 1, 2, 3 and 4 on the vertical coordinate represent the four operating conditions of the gearbox: NOR; BTF; MTF; and SWF.

The diagnostic accuracies of the six entropy methods are as follows: 98.33%; 90%; 96.67%; 96.67%; 96.67%; and 91.67%, respectively, based on the results presented in Figure 14. MHFDE_TANSIG has the highest accuracy of all entropy models. It can be seen that none of the six entropy algorithms misclassify BTF or classify other faults as BTF from the distribution of misclassifications in the diagnostic results, indicating that BTF can be fully distinguished from other faults in the feature set of the entropy algorithms. The remaining misclassifications are all concentrated between MTF and SWF except that the first three entropy methods all misclassify NOR samples into MTF at one time, while only MHFDE_TANSIG shows no misclassification between these two types of defect states. It can be seen that the proposed method is superior to other entropy methods and can effectively discriminate different fault states of gearboxes.

**Figure 15:**
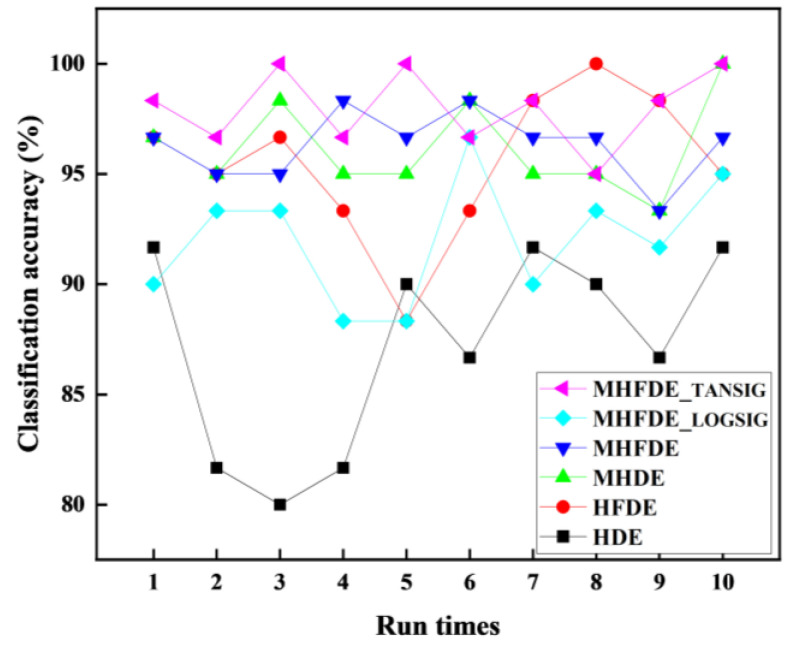
The diagnostic results of multiple classifications.

The same fault characteristics extracted in Section 3.2 were entered into the NGO–SVM model to identify faults ten times, with the objective of evaluating the performance of the intelligent diagnostic method and to prevent random interference. Figure 15 illustrates the diagnostic outcomes of multiple classifications, while Table 9 provides the maximum, minimum, and average accuracy rates.

The MHFDE_TANSIG intelligent diagnostic model’s maximum, minimum, and average accuracies are 100%, 95%, and 98%, respectively, according to Table 9. These accuracies of the MHFDE_TANSIG intelligent diagnostic model are higher than other intelligent diagnostic models, proving the superiority of this intelligent diagnostic model.

### 3.4. Public Gearbox Datasets

We also selected the Southeast University (SEU) Gear Dataset and the University of Connecticut (UConn) Gear Dataset to confirm the superiority of the proposed intelligent diagnostic model in addition to the gearbox dataset that we collected ourselves from the mechanical failure simulation (MFS) experiment system.

#### 3.4.1. Southeast University Gear Dataset

The gearbox dataset was acquired from Southeast University, China. The data were acquired from the Driveline Dynamic Simulator, which is a test rig comprising a motor, a motor controller, a planetary gearbox, a reduction gearbox, a brake, and a brake controller [42]. The test module was fitted with gears exhibiting various failure conditions to generate experimental data.

The motor speed was set to 1800 rpm, and the vibration indications obtained from the sensors along the y-axis on the planetary gear were chosen to run with a load of 7.32 Nm during the experiment. The SEU Gear Dataset tested five operating conditions: healthy tooth; chipped tooth; missing tooth; surface fault; and root fault. There are 375 samples in the dataset, divided into 5 groups, and each group contains 75 subsamples of 2048 sampling points each. The set of samples for each fault state is segregated into two categories: 60 samples designated for training and 15 for testing in fault diagnosis scenarios. (See Table 10).

**Figure 16:**
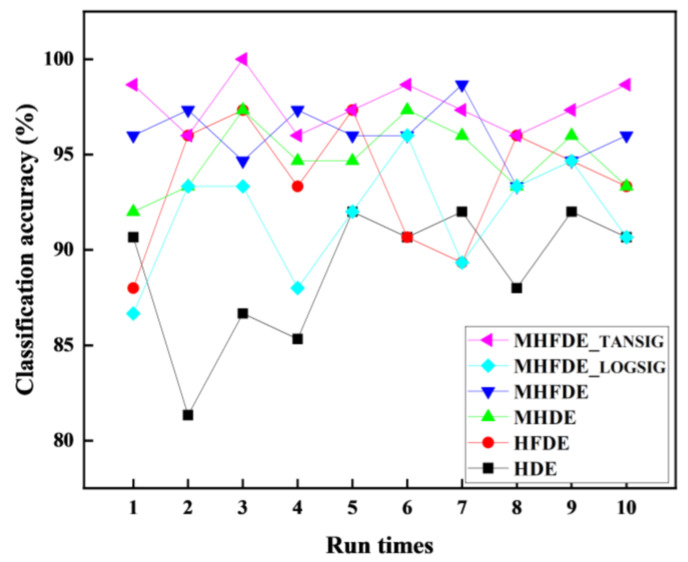
The diagnostic results of multiple classifications on SEU Data.

The MHFDE_TANSIG value and other entropy values were calculated for the 375-sample SEU Gear Dataset. The same fault characteristics were then entered into the NGO–SVM model to identify faults ten times. In Figure 16 and Table 11, the diagnostic results of multiple classifications are shown.

According to Table 11, the MHFDE_TANSIG intelligent diagnostic model’s maximum, minimum, and average accuracies are 100%, 96%, and 97.6%, respectively, which are higher than other intelligent diagnostic models.

#### 3.4.2. University of Connecticut Gear Dataset

This section of the experiment uses the experimental dataset of gearbox vibration experiments from the University of Connecticut. The experimental apparatus comprises a two-stage reference gearbox, which includes gears on the input shaft of the first stage and the output shaft of the second stage. The magnetic brake is regulated by varying its input voltage. The vibration signals were captured with a 20 kHz sampling frequency using a dSPACE system [43]. 

Simulated failure states were introduced for the gear of the first stage. The UConn Gear Dataset tested nine different gear work states, comprising five failure types (health, missing tooth, root crack, spalling, and chipping faults) and five wear levels (five different severities of chipping faults). There are 900 samples in the dataset, divided into 9 groups, and each group contains 100 subsamples of 2048 sampling points each. The set of samples for each fault state is segregated into two categories: 70 samples designated for training and 30 for testing in fault diagnosis scenarios. (See Table 12).

**Figure 17:**
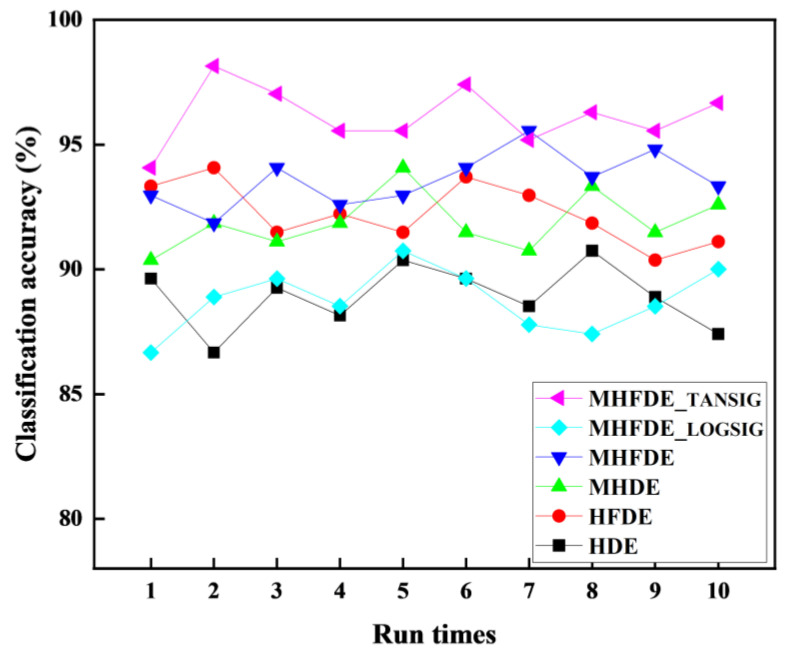
The diagnostic results of multiple classifications on UConn Data.

The MHFDE_TANSIG value and other entropy values were calculated for the 900-sample UConn Gearbox Dataset. The same fault characteristics were then entered into the NGO–SVM model to identify faults ten times. In Figure 17 and Table 13, the diagnostic results of multiple classifications are shown.

According to Table 13, the MHFDE_TANSIG intelligent diagnostic model’s maximum, minimum, and average accuracies are 98.15%, 94.07%, and 96.15%, respectively, which are higher than other intelligent diagnostic models.

### 3.5. Result Analysis

**Figure 18:**
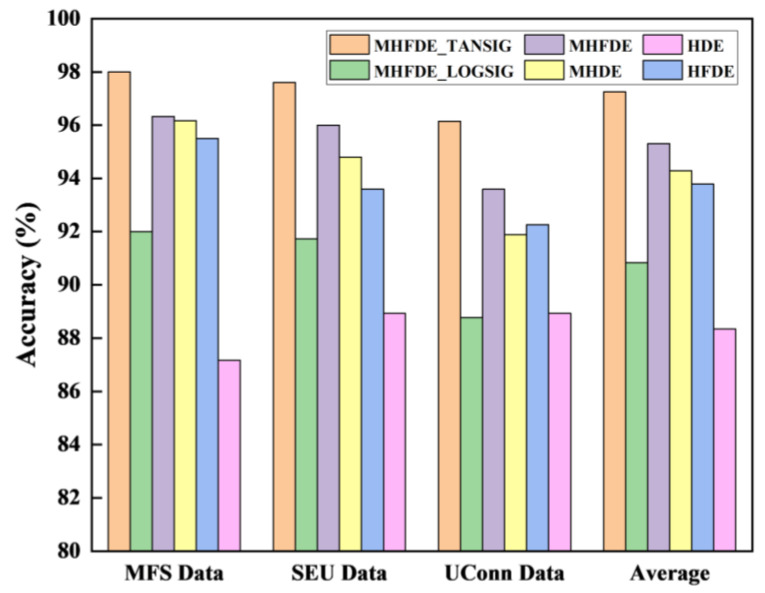
Accuracy of different entropy methods.

We record the average accuracy of different entropy algorithms for diagnosis under each of the three datasets (MFS Data, SEU Data, and UConn Data), as well as the average of each type of entropy algorithm recording the average accuracy under each of the three different data sources in Figure 18.

The accuracy of HFDE surpasses that of HDE, and MHFDE exhibits a greater degree of accuracy than MHDE. This suggests that the fluctuation-based DE method outperforms the traditional DE method. The reason for this is that the FDE considers the relative relationship between signals, rather than the absolute relationship of the traditional DE.

The accuracy of recognition is higher in MHFDE than in HFDE, and in MHDE than in HDE. This suggests that the modified hierarchical decomposition outperforms the traditional hierarchical decomposition. The improved method effectively addresses the disadvantage of the unimproved hierarchical decomposition, which is the diminution in length after layering.

MHFDE_TANSIG has higher recognition accuracy than MHFDE_LOGSIG and MHFDE, indicating that the different mapped MHFDEs are inconsistent in distinguishing gearbox conditions and the TANSIG mapping is the most effective. This is because TANSIG has better noise immunity than the other two mapping methods.

Taken together, it is evident that MHFDE_TANSIG efficiently extracts the fault features of various gearbox states, and the performance of its feature extraction is preferable to other comparative methods.

## 4. Conclusions

This paper presented a novel fault diagnosis model based on MHFDE_TANSIG and NGO–SVM, which was then applied to gearbox test data. The entropy method was employed to extract features from gearbox fault data, which was then combined with machine learning techniques to resolve the issue of identifying wind turbine gearbox faults via vibration signals. The following conclusions were reached:

1. The MHFDE_TANSIG diagnostic model was found to have higher classification accuracy than MHFDE_LOGSIG, MHFDE, MHDE, HFDE, and MDE from the fault diagnosis results of the gearbox vibration datasets. This demonstrates the effectiveness and superiority of the improved entropy algorithm in gearbox fault diagnosis.
2. The data source chosen for the experiments in this paper is a gearbox vibration dataset that includes three different sources, and several repetitive experiments were conducted to obtain a high average recognition accuracy. The experimental results demonstrate the stability and generalization of the proposed diagnostic model.
3. The experimental results indicate that the average identification accuracy of the MHFDE_TANSIG diagnostic model for gearbox faults is 97.25%. This provides a new method for the fault diagnosis of gearboxes and also offers a novel approach to fault diagnosis in the field of rotating machinery.
4. In this paper, the main application for the state analysis of wind turbine gearboxes is acceleration sensor information, which will lack the accuracy of the comprehensive assessment of the gearbox operating state. Consequently, it is essential to integrate the vibration, temperature, current, and voltage signals within the existing monitoring system in order to enhance the assessment of operating conditions. This will facilitate the generation of more accurate and comprehensive results. Further research could be conducted from the perspective of information fusion of multiple monitoring signals, utilizing the complementary characteristics of different sensor signals to analyze the operating characteristics of the gearbox, thereby facilitating fault monitoring.

## Figures and Tables

**Table 1:** Running time for different *k*.

Types	Time (s)				
	*k* = 1	*k* = 2	*k* = 3	*k* = 4	*k* = 5
WGN	0.1060	0.2509	0.4522	1.2964	2.0964
1/f noise	0.0946	0.3488	0.4682	0.9868	2.2760

**Table 2:** Running time for different *l*.

Types	Time (s)				
	*l* = 8192	*l* = 4096	*l* = 2048	*l* = 1024	*l* = 512
WGN	1.0398	0.7033	0.4681	0.4488	0.3626
1/f noise	0.9783	0.6958	0.4810	0.4006	0.3793

**Table 3:** CV for different *l*.

Types	CV				
	*l* = 8196	*l* = 4096	*l* = 2048	*l* = 1024	*l* = 512
WGN	0.0439	0.0317	0.0239	0.0187	0.0125
1/f noise	0.0551	0.0303	0.0276	0.0258	0.0148

**Table 4:** Running time for different *d*.

Types	Time (s)				
	*d* = 1	*d* = 2	*d* = 3	*d* = 4	*d* = 5
WGN	0.4548	0.4735	0.4621	0.4634	0.4550
1/f noise	0.4639	0.4635	0.4701	0.4635	0.4687

**Table 5:** Running time for different *m*.

Types	Time (s)				
	*m* = 2	*m* = 3	*m* = 4	*m* = 5	*m* = 6
white	0.4801	0.7227	1.9838	11.4546	109.5944
1/f	0.4888	0.6912	2.0941	11.3713	125.9975

**Table 6:** Running time for different *c*.

Types	Time (s)				
	*c* = 3	*c* = 4	*c* = 5	*c* = 6	*c* = 7
white	0.4598	0.4509	0.4760	0.4781	0.4763
1/f	0.4669	0.4782	0.4860	0.4762	0.4973

**Table 7:** Parameters and mapping function of different entropy algorithms.

Entropy Methods	Mapping Function	Parameters
MHFDE_TANSIG	TANSIG	*l* = 2048, *k* = 3, *d* = 1, *m* = 2, *c* = 5
MHFDE_LOGSIG	LOGSIG	*l* = 2048, *k* = 3, *d* = 1, *m* = 2, *c* = 5
MHFDE	NCDF	*l* = 2048, *k* = 3, *d* = 1, *m* = 2, *c* = 5
HFDE	NCDF	*l* = 2048, *k* = 3, *d* = 1, *m* = 2, *c* = 5
MHDE	NCDF	*l* = 2048, *k* = 3, *d* = 1, *m* = 2, *c* = 5
HDE	NCDF	*l* = 2048, *k* = 3, *d* = 1, *m* = 2, *c* = 5

**Table 8:** Description of the MFS gearbox dataset.

Types	Number of Training Sets	Number of Prediction Sets	Working Condition (Speed—Load)
Normal	35	15	1750 rpm—0 Nm
Broken tooth	35	15	1750 rpm—0 Nm
Missing tooth	35	15	1750 rpm—0 Nm
Surface wear	35	15	1750 rpm—0 Nm
**Totality**	140	60	1750 rpm—0 Nm

**Table 9:** Diagnostic accuracy of intelligent diagnostic models.

Entropy Value	Accuracy (%)		
	Maximum	Minimum	Average
MHFDE_TANSIG	100.00	95.00	98.00
MHFDE_LOGSIG	96.67	88.33	92.00
MHFDE	98.33	93.33	96.33
MHDE	100.00	93.33	96.17
HFDE	100.00	88.33	95.50
HDE	91.67	80.00	87.17

**Table 10:** Description of the SEU gearbox dataset.

Types	Number of Training Sets	Number of Prediction Sets	Working Condition (Speed—Load)
Healthy tooth	60	15	1800 rpm—7.32 Nm
Chipped tooth	60	15	1800 rpm—7.32 Nm
Missing tooth	60	15	1800 rpm—7.32 Nm
Surface fault	60	15	1800 rpm—7.32 Nm
Root fault	60	15	1800 rpm—7.32 Nm
**Totality**	300	75	1800 rpm—7.32 Nm

**Table 11:** Diagnostic accuracy of intelligent diagnostic models on SEU Data.

Entropy Value	Accuracy (%)		
	Maximum	Minimum	Average
MHFDE_TANSIG	100.00	96.00	97.60
MHFDE_LOGSIG	96.00	86.67	91.73
MHFDE	98.67	93.33	96.00
MHDE	97.33	92.00	94.80
HFDE	97.33	88.00	93.60
HDE	92.00	81.33	88.93

**Table 12:** Description of the UConn gearbox dataset.

Types	Number of Training Sets	Number of Prediction Sets
Health	70	30
Missing tooth	70	30
Root crack	70	30
Spalling	70	30
Chipping tip_5	70	30
Chipping tip_4	70	30
Chipping tip_3	70	30
Chipping tip_2	70	30
Chipping tip_1	70	30
**Totality**	630	270

**Table 13:** Diagnostic accuracy of intelligent diagnostic models on UConn Data.

Entropy Value	Accuracy (%)		
	Maximum	Minimum	Average
MHFDE_TANSIG	98.15	94.07	96.15
MHFDE_LOGSIG	90.74	86.67	88.78
MHFDE	95.56	91.85	93.59
MHDE	94.07	90.37	91.89
HFDE	94.07	90.37	92.26
HDE	90.74	86.67	88.93

## Data Availability

The data presented in this study are available on request from the corresponding author.

## Abbreviations

The following abbreviations are used in this manuscript:

DE	Dispersion entropy
FDE	Fluctuation dispersion entropy
HDE	Hierarchical dispersion entropy
HFDE	Hierarchical fluctuation dispersion entropy
MHDE	Modified hierarchical dispersion entropy
MHFDE	Modified hierarchical fluctuation dispersion entropy
TANSIG	Tan-sigmoid
LOGSIG	Log-sigmoid
MHFDE_LOGSIG	MHFDE of LOGSIG mapping
MHFDE_TANSIG	MHFDE of TANSIG mapping
NCDF	Normal cumulative distribution function
WGN	White Gaussian noise
CV	Coefficient of variation
NOR	Normal state
MTF	Missing tooth fault
BTF	Broken tooth fault
SWF	Surface wear fault
PSO	Particle swarm optimization
GA	Genetic algorithm
NGO	Northern goshawk optimization
SVM	Support vector machine
NGO–SVM	NGO algorithm to optimize the SVM
MFS	Mechanical failure simulation
SEU	Southeast University Gear Dataset
UConn	University of Connecticut
