# Correction: Wang, X.; Du, Y. Fault Diagnosis of Wind Turbine Gearbox Based on Modified Hierarchical Fluctuation Dispersion Entropy of Tan-Sigmoid Mapping. *Entropy* 2024, *26*, 507

**DOI:** 10.3390/e26090733

**Published:** 2024-08-29

**Authors:** Xiang Wang, Yang Du

**Affiliations:** 1 School of Energy and Power Engineering, Nanjing Institute of Technology, Nanjing 211167, China; wangxiang@njit.edu.cn; 2 School of Electrical Engineering, Nanjing Institute of Technology, Nanjing 211167, China

In the original publication [1], the dataset of the Mechanical Failure Simulation Experiment System (MFS) gearbox for 1750 rpm was erroneously labelled as 1200 rpm. Corrections have been made as follows:

Section 3.1, Paragraph 2

 

The motor speed was set to 1750 rpm, and the vibration indications obtained from the sensors along the y-axis on the planetary gear were chosen to run under no load during the experiment. There were four operating conditions tested: NOR; MTF; BTF; and SWF. There are 200 samples in the dataset divided into 4 groups, and each group contains 50 subsamples of 2048 sampling points each. The set of samples for each fault state is segregated into two categories: 35 samples designated for training and 15 for testing in fault diagnosis scenarios (see Table 8). Figure 11 illustrates the waveforms of the vibration indications of the gearbox under four distinct operating conditions. The horizontal coordinate indicates the duration of the captured clip in seconds s, and the vertical coordinate shows the vibration acceleration of the gearbox in the unit of gravity acceleration g in Figure 11.

 

The corrected Table 8 appears below.

**Table 8:** Description of the MFS gearbox dataset.

Types	Number of Training Sets	Number of Prediction Sets	Working Condition (Speed—Load)
Normal	35	15	1750 rpm—0 Nm
Broken tooth	35	15	1750 rpm—0 Nm
Missing tooth	35	15	1750 rpm—0 Nm
Surface wear	35	15	1750 rpm—0 Nm
**Totality**	140	60	1750 rpm—0 Nm

The authors state that the scientific conclusions are unaffected. This correction was approved by the Academic Editor. The original publication [1] has also been updated.
